# Precision matters: Repeatability and reproducibility of total PSA and homocysteine measurements in Alinity i-system

**DOI:** 10.5937/jomb0-48306

**Published:** 2024-06-15

**Authors:** Oussama Grari, Amina Himri, Nisma Douzi, Soufiane Beyyoudh, Imad-Eddine Elkhamlichi, Kaoutar Benaissa, Dounia Elmoujtahide, El-Houcine Sebbar, Mohammed Choukri

**Affiliations:** 1 Mohammed I University, Faculty of Medicine and Pharmacy, Oujda, Morocco; 2 Mohammed VI University Hospital, Department of Biochemistry, Oujda, Morocco

**Keywords:** method verification, precision, CMIA, PSA, homocysteine, verifikacija metode, preciznost, CMIA, PSA, homocistein

## Abstract

**Background:**

The precision of test measurements is critical in clinical diagnostics, especially for biomarkers like total PSA and homocysteine, which are essential to disease assessment. Using the CMIA approach, this study investigates the repeatability and reproducibility of these biomarkers on the Abbott Alinity system.

**Methods:**

The present study was conducted in the clinical chemistry laboratory at Mohammed VI University Hospital of Oujda. The evaluation of the Alinity i-system's analytical performance for total PSA and homocysteine focused on assessing repeatability and intermediate precision. The assessment followed the protocols and guidelines established by the French Accreditation Committee (COFRAC).

**Results:**

Our analysis yielded favorable findings regarding the performance of the Alinity assays. The coefficients of variation for both the within-run and between-run precision were less than 5.89% and 4.29%, respectively. These findings produce acceptable outcomes compared to the manufacturer's claims and the SFBC database. Our study underscores the tests' precision, affirming the CMIA method's reliability in measuring total PSA and homocysteine levels.

**Conclusions:**

The assessment of the Alinity i-system for total PSA and homocysteine showed significant analytical performance. Our findings have implications for laboratory personnel, researchers, and physicians supporting a continuous diagnostic accuracy improvement culture.

## Abstract (sr)

**Uvod:**

Preciznost merenja testova je ključna u kliničkoj dijagnostici, posebno za biomarkere kao što su ukupni PSA i homocistein, koji su bitni za procenu bolesti. Koriste i CMIA pristup, ova studija istražuje ponovljivost i reproduktibilnost ovih biomarkera na sistemu Abbott Alinity.

**Metode:**

Ova studija je sprovedena u kliničko-hemijskoj laboratoriji bolnice Muhamed VI u Uždi, Maroko. Evaluacija analitičke performanse Alinity i-sistema za ukupni PSA i homocistein je bila fokusirana na procenu ponovljivosti i međusobne preciznosti. Procena je pratila protokole i smernice koje je uspostavio Francuski akreditacioni komitet (COFRAC).

**Rezultati:**

Naša analiza je dala povoljne nalaze u vezi sa performansama Alinity testova. Koeficijenti varijacije za preciznost unutar serije i između serija su bili manji od 5,89% odnosno 4,29%. Ovi nalazi daju prihvatljive rezultate u poređenju sa tvrdnjama proizvođača i SFBC bazom podataka. Naša studija u prvi plan stavlja preciznost testova, potvrđuju i pouzdanost CMIA metode pri merenju nivoa ukupnog PSA i homocisteina.

**Zaključak:**

Procena Alinity i-sistema za ukupni PSA i homocistein je pokazala značajne analitičke performanse. Naši nalazi imaju implikacije za osoblje laboratorija, istraživače i lekare i u skladu su sa kulturom neprekidnog poboljšanja tačnosti dijagnostike.

## Introduction

Monitoring and maintaining quality throughout the testing process – including pre-analytical, analytical, and post-analytical stages – is the core concept of quality assurance. Currently, laboratories worldwide rely on manufacturer-validated techniques [1]. However, in adherence to ISO 15189:2022 standards, these validated methods must undergo independent laboratory verification before becoming an integral part of routine practice [2]. In our study, we performed method verification of two parameters to ascertain adherence to the specified standards.

Prostate-specific antigen (PSA) is a serine protease enzyme the prostatic tissue generates. Total PSA (tPSA) and free PSA have been utilized as diagnostic indicators for screening and monitoring treatment response for prostate cancer patients. In addition to prostate cancer, other noncancerous conditions, such as prostatitis and benign prostate hyperplasia, can also cause tPSA elevations [3].

Homocysteine (Hcy), an amino acid containing sulfur, is generated during the demethylation process of methionine, an essential amino acid found in food proteins [4]. Hyperhomocysteinemia is an increased concentration of Hcy in the bloodstream, which can be attributed to various environmental and genetic causes [5]. Elevated levels of Hcy have been linked to cardiovascular complications, neurodegeneration, diabetes, Down syndrome, neural tube defects, megaloblastic anemia, and cancer [6].

Several assays are available for measuring Hcy and tPSA, with commercial options relying predominantly on immunoassays.

As part of our efforts to advance knowledge about diagnostic testing platforms, we want to offer a comprehensive understanding of the precision associated with tPSA and Hcy assays using the Abbott Alinity i-System (Abbott Laboratories) following the French Accreditation Committee (COFRAC) guidelines. Our research aims to increase confidence in diagnostic accuracy by analyzing the precision of this modern technology, providing crucial insights into the dependability of these assays in clinical situations.

## Materials and methods

### Study design

The present investigation was conducted in the clinical chemistry laboratory affiliated with Mohammed VI University Hospital in Oujda. The tPSA and Hcy assays were run on an Alinity analyzer to assess repeatability and intermediate precision.

Scope A of the SH GTA 04, issued by the French Accreditation Committee (COFRAC), was adopted.

Following the manufacturer’s guidelines, the controls were analyzed after calibrating the assay. The outcomes obtained from the control materials were to the manufacturer’s specifications. We utilized Multichem IA Quality Control material (Technopath Clinical Diagnostics) to evaluate repeatability. Every level of control material was tested thirty times. The intermediate precision was assessed over thirty days using daily control samples of low, medium, and high concentrations. Mean concentration, standard deviation, and coefficient of variation were calculated for the two parameters.

### Analysis

The data processing was executed using the EVM Middleware, developed by BYG Informatique.

### Principles of the test technique

### Total PSA

Using chemiluminescent microparticle immunoassay (CMIA) technology, the Alinity i tPSA assay is a two-step immunoassay for the quantitative determination of tPSA (both free PSA and PSA complexed to alpha-1-antichymotrypsin) in human serum. Paramagnetic microparticles coated with anti-PSA attach to the PSA in the sample. After washing, a conjugate labeled with anti-PSA acridinium is added to the reaction mixture. After an additional washing cycle, the reaction mixture is treated with pre-trigger and trigger solutions. The resultant chemiluminescent reaction is measured in relative light units (RLU). The RLU determined by the Alinty System optics correlate with the sample’s tPSA concentration [7].

### Homocysteine

The Alinity Hcy assay uses CMIA technology in a single step to quantify total L-homocysteine in human serum or plasma. Free Hcy is produced when dithiothreitol reduces bound or dimerized Hcy. In the presence of excess adenosine, this free Hcy is converted to S-adenosyl-homocysteine (SAH) via the recombinant enzyme S-adenosyl-L-homocysteine-hydrolase (SAHH). Acridinium-labeled S-adenosyl cysteine then competes with the SAH for the particle-bound monoclonal antibody. After a wash step and magnetic separation, the reaction mixture is supplemented with pre-trigger and trigger solutions. The chemiluminescence that results is subsequently quantified in relative light units (RLU). The amount of Hcy in the sample and the RLU detected by the Alinity i-System optics are related indirectly [7].

## Results

### Repeatability

Estimating repeatability (within-run precision) requires repeated measurements using an identical measuring procedure, operators, analyzing system, operating environment, and location on the same or similar objects within a short period [8].

### Intermediate precision

Intermediate precision, also called between-run precision, is assessed by conducting replicate measurements on the same sample under conditions of the same measurement procedure and location while varying some operating conditions, including time, operator, calibration, and equipment [9] [10]. Table 1 and Table 2 present the mean, standard deviation, and percentage coefficient of variation values for the tPSA and Hcy assays.

**Table 1:** Results of precision for tPSA assay. SD, Standard deviation; CV, coefficient of variation

	Level 1	Level 2	Level 3
Repeatability (n=30)			
Mean (μg/L)	0.67	3.31	22.73
SD (μg/L)	0.021	0.139	1.339
CV (%)	3.22	4.21	5.89
Intermediate precision (n=30)			
Mean (μg/L)	0.66	3.25	21.32
SD (μg/L)	0.024	0.139	0.902
CV (%)	3.69	4.29	4.23

**Table 2:** Results of precision for Hcy assay. SD, Standard deviation; CV, coefficient of variation

	Level 1	Level 2	Level 3
Repeatability (n=30)			
Mean (μmol/L)	10.92	16.45	23.36
SD (μmol/L)	0.304	0.474	0.638
CV (%)	2.78	2.88	2.73
Intermediate precision (n=30)			
Mean (μmol/L)	11.60	15.61	23.98
SD (μmol/L)	0.375	0.500	0.635
CV (%)	3.23	3.20	2.65

## Discussion

Routinely, serum PSA testing is utilized to diagnose prostate cancer and track the progression of the disease. A tPSA level exceeding 4.0 ng/mL has a predictive value for prostate cancer and indicates the need for a biopsy. Furthermore, when comparing men with prostate cancer to men with benign prostatic hyperplasia, free PSA levels were considerably lower in proportion to tPSA [11]. Today, accessible commercial assays utilize the immunochemical quantification of circulating free and complexed to α1-antichymotrypsin forms of PSA to determine tPSA and free PSA [12].

Hcy, a sulfur amino acid produced during methionine metabolism, can be a biomarker and a prognostic indicator for cardiovascular events and mortality at high concentrations (Hcy>13–15 μmol/L). Various analytical methods are available to determine Hcy, with immunoassays commonly employed in diagnostic settings. These include enzyme immunoassay (EIA), fluorescence polarization immunoassay (FPIA), and chemiluminescence immunoassay (CLIA) [13].

Immunoassays possess elevated levels of sensitivity and specificity, as well as substantial throughput. It is suitable for quantifying diverse analytes that prove challenging to ascertain via alternative analytical techniques [14]. As a result, it has found extensive application in the quantification of drug concentration, monitoring of therapeutic drugs, analysis of hormones, and detection of disease-specific proteins such as tumor and cardiac injury markers [15].

Precision is the degree of agreement between replicate measurements performed on the same or similar objects under specified conditions [8]. Typically, precision is not calculated; instead, it is quantitatively described in terms of imprecision, and this includes measuring the standard deviation and coefficient of variation of the results over a series of measurement repetitions [10].

Assessing precision is a step in validating or verifying a method to ensure its appropriateness for use. Clause 7.3.2 of ISO 15189:2022 states that the laboratory must have a procedure to verify examination methods before using them, ensuring that the manufacturer’s or method’s specified performance can be satisfied [2].

Precision evaluation includes measurement of repeatability and intermediate precision. Guidelines for this process exist, such as SH GTA 04 from the French Committee of Accreditation (COFRAC) and EP15-A3 from the Clinical and Laboratory Standards Institute (CLSI).

Our study used the COFRAC guide SH GTA 04 to assess the precision of the Abbott tPSA and Hcy assays. Analysis of the CV of repeatability revealed distinct patterns for the tPSA and Hcy assays. For tPSA, our CV values were in concordance with the SFBC database [16], meeting their specified benchmarks. However, they exhibited a slight elevation compared to the manufacturer’s claims, except for the first level, which aligned with the manufacturer’s specifications. Meanwhile, the Hcy assay presented a unique challenge, as no CV reference values were available in the SFBC database. In our comparison with the manufacturer’s claims, all the CV values for the three assay levels were higher than expected. This discrepancy seems to originate from the different aspects of the manufacturer’s facility, a strictly controlled setting, and the real-world challenges of a typical clinical laboratory. On the other hand, all of the CV values in our investigation were acceptable for intermediate precision. Table 3 and Table 4 compare our study’s CV with the SFBC database and manufacturer’s claims.

**Table 3:** Repeatability CV Comparison for tPSA and Hcy Measurements. NA. not available; SFBC. French Society of Clinical Biology

		Our study	SFBC (16)	Manufacturer
tPSA	Level 1	3.22	18.17	3.5
	Level 2	4.21	6.36	3.9
	Level 3	5.89	6.36	4.3
Hcy	Level 1	2.78	*NA*	1.7
	Level 2	2.88	*NA*	1.8
	Level 3	2.73	*NA*	1.2

**Table 4:** Intermediate precision CV Comparison for tPSA and Hcy Measurements.

		Our study	SFBC (16)	Manufacturer<br>claims
tPSA	Level 1	3.69	24.16	4.1
	Level 2	4.29	8.48	4.4
	Level 3	4.23	8.46	4.7
Hcy	Level 1	3.23	*NA*	5.6
	Level 2	3.20	*NA*	5.1
	Level 3	2.65	*NA*	4.3

Although this study was conducted rigorously, significant limitations should be addressed. The primary focus was on repeatability and intermediate precision, with key metrics like linearity, method comparability, and measurement uncertainty excluded. Furthermore, various external elements that could affect the method’s performance may not have been adequately considered. Because this was a single-center investigation, potential imprecision resulting from inter-laboratory discrepancies in a multicenter study environment was not considered.

## Conclusion

In conclusion, the Alinity i-system evaluation demonstrated significant analytical performance, notably good precision in our assessment. However, it is critical to recognize the study’s deficiencies. The lack of essential parameters such as linearity, method comparison, and measurement uncertainty limits a thorough understanding of the system’s overall performance.Addressing such limitations in future research efforts will be critical in giving a more comprehensive and trustworthy evaluation of the Alinity i-system’s capabilities across various parameters and settings.

## Dodatak

### Supplementary data

Supplementary data will be accessible on demand.

### Conflict of interest statement

All the authors declare that they have no conflict of interest in this work.

### List of Abbreviations

PSA, prostate-specific antigen;<br>tPSA, total PSA;<br>Hcy, homocysteine;<br>CMIA, chemiluminescent microparticle immunoassay;<br>SFBC, French Society of Clinical Biology;<br>SAH, S-adenosyl homocysteine;<br>RLU, relative light units;<br>SAAH, S-adenosyl-L-homocysteine hydrolase.
